# Congenital Transmesenteric Hernia—Importance of a Timely Intervention

**DOI:** 10.1055/s-0040-1710533

**Published:** 2020-06-19

**Authors:** Mandar Sharadchandra Koranne, Amay Banker

**Affiliations:** 1 Department of General Surgery, Seth GS Medical College and KEM Hospital, Mumbai, Maharashtra, India

**Keywords:** transmesenteric hernia, superior mesenteric vein compression

## Abstract

Transmesenteric hernia is a rare cause of small bowel strangulation in adults. Our patient was a 61-year-old previously healthy male, who presented with vomiting and abdominal pain with no surgical history and no trauma in the past. Computed tomography with contrast enhancement was suggestive of superior mesenteric vein (SMV) compression without any obvious cause. The emergency exploratory laparotomy revealed venous congestion of small bowel caused by a transmesenteric hernia with the herniated loop compressing the SMV. On reducing the hernia, complete reversal of the bowel congestion was noted and small bowel resection was averted. A high index of suspicion for a transmesenteric hernia in small bowel obstruction of unknown etiology and a timely surgical intervention are must for a good clinical outcome.

Internal hernias are a rare cause of intestinal obstruction. The diagnosis is often elusive even in its acute state and is usually diagnosed intraoperatively. There are many case reports suggestive of a congenital mesenteric defect in the pediatric population. Here we present a case of a congenital internal hernia in a previously healthy 61-year-old male with the herniated bowel loop causing compression of the superior mesenteric vein (SMV). On reducing the hernia, entire dusky small bowel reverted to a normal pink color. The mesenteric defect was closed and the patient was discharged with an uneventful postoperative course.

## Case Report

A 61-year-old male patient presented to our emergency with a 15-day history of constipation and multiple episodes of nonbilious vomiting. He had no surgical history, no comorbidities, and denied any abdominal trauma in the past. The patient was evaluated elsewhere and was given a trial of conservative management. He had undergone a contrast-enhanced computed tomography (CECT) scan of abdomen and pelvis that revealed 1.5 L of ascites which was hemorrhagic on tap. The patient was conscious, oriented, and afebrile on admission with a pulse of 100 beats per minute and blood pressure of 120/86 mm Hg. Physical examination revealed a soft but distended abdomen that was not tender on palpation. Per rectal examination revealed an empty rectum and no stains on returning finger.

**Fig. 1:**
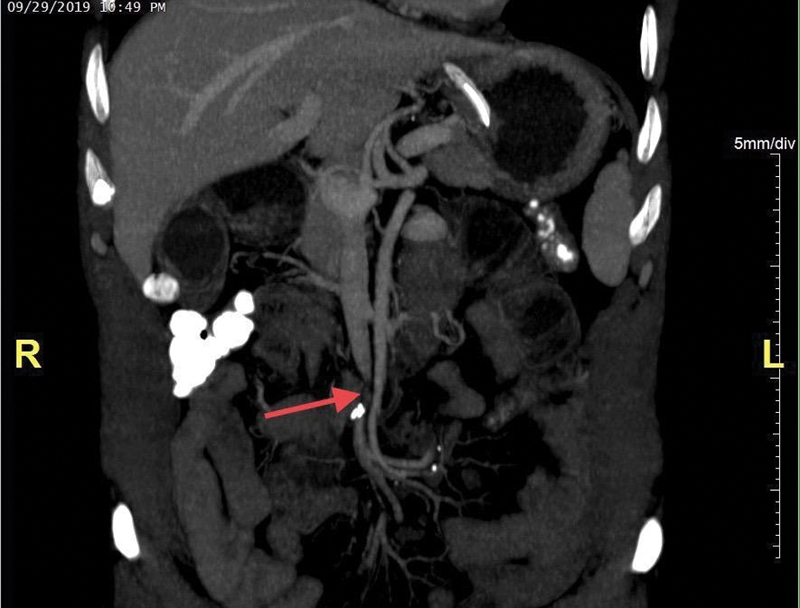
Red arrow showing superior mesenteric vein compression. No other signs of small bowel obstruction, ischemic bowel, ascites, malignancy, or internal herniation can be seen.

All laboratory parameters were within normal limits. No air fluid levels were seen on an abdominal X-ray in erect position. A repeat CECT of abdomen and pelvis revealed no signs of intestinal obstruction, ischemic bowel disease, ascites, or malignancy. However, compression of SMV was seen ( Fig. 1 ). A nasogastric tube was inserted that drained 700 mL of bilious output within 1 hour. In view of the above findings, decision was taken to explore the patient.

**Fig. 2:**
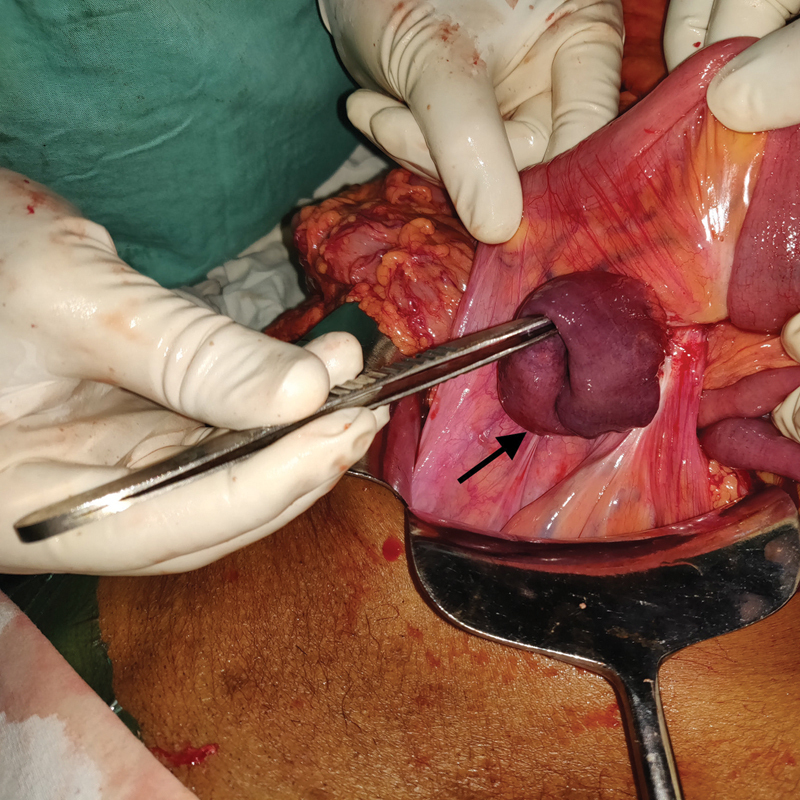
A transmesenteric hernia with dusky small bowel loop herniating through the defect. The black arrow shows the mesenteric defect with a dusky terminal ileal loop herniating through it.

**Fig. 3:**
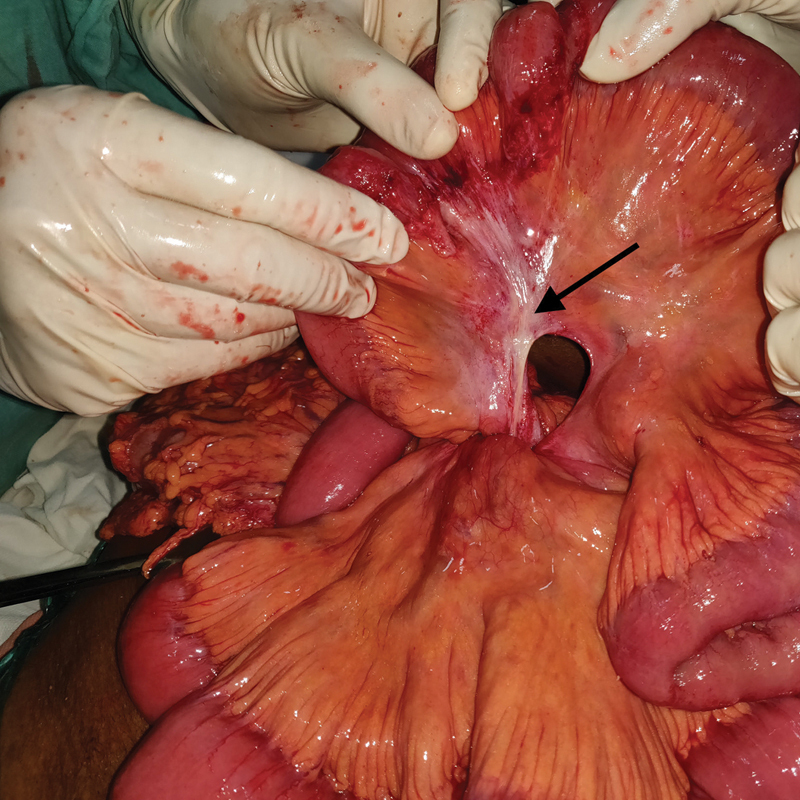
A congenital transmesenteric defect with fibrotic edges and no surrounding fibrosis as shown by the black arrow. Complete reversal of bowel color can be seen.

**Fig. 4:**
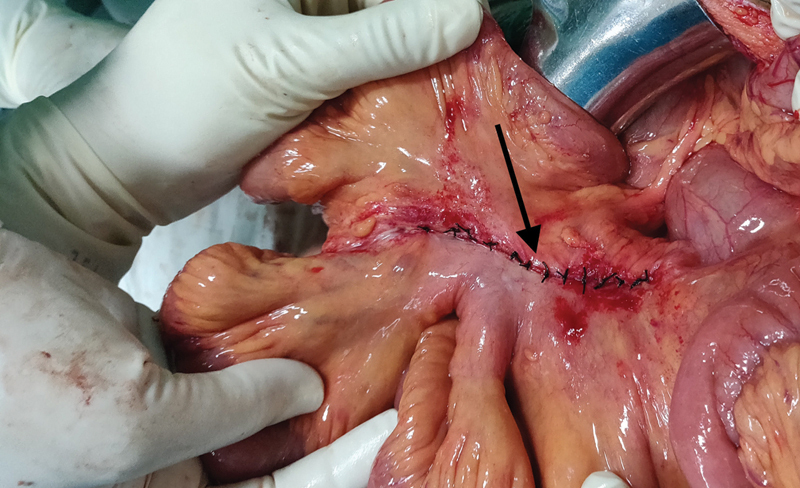
The mesenteric defect closed with interrupted 3–0 silk sutures.

We observed 15 cm of dusky bowel herniating through a 10 cm × 6 cm mesenteric defect in the terminal ileum ( Fig. 2 ). The entire bowel in the SMV territory was dusky in appearance. The edge of the defect was fibrotic indicating a long-standing nature of the defect. No adhesions were seen near the defect ( Fig. 3 ). No blood or free fluid was present in the peritoneal cavity. On reduction in the herniated bowel loop, complete reversal of the bowel color was noted. Viability of the bowel loops was confirmed and the mesenteric defect was closed with 3–0 nonabsorbable interrupted sutures ( Fig. 4 ).

The postoperative course of the patient was uneventful. Patient was gradually weaned on to full diet by postoperative day 4 and was discharged 8 days after the surgery.

## Discussion

Internal hernias, deﬁned as a protrusion of viscus through an intra-abdominal aperture without traversing fascial planes, are a rare cause of intestinal obstructions. These consist of paraduodenal (53%), pericecal (13%), foramen of Winslow (8%), transmesenteric and transmesocolic (8%), intersigmoid (6%), and retroanastomotic (5%) hernias. 1

Although transmesenteric hernias were previously uncommon, a rise in Roux-en-Y bypass and liver transplant surgeries have led to an increase in their incidence. In the pediatric population, these defects are commonly congenital and in the region of the small bowel (70% of cases), with 53% of these being in the ileocecal area of the mesentery. 2 This area was originally described by Treves in 1885. He noted an area of the mesentery (later named the Treves' Field) circumscribed by the last ileal branch and the ileocolic artery that contained no fat, no lymph nodes, and no visible vessels rendering it highly susceptible to injury during development. 3 Other possible etiologies of a mesenteric defect include intraperitoneal inflammation, trauma, partial developmental regression, and fenestration of the mesentery by the colon during embryologic displacement into the umbilical cord. 1

In adults, these defects are usually acquired as a result of either blunt abdominal trauma or surgical manipulation of the bowel and mesentery. Congenital mesenteric defects are very rare in adults but can cause an internal hernia followed by an incarceration or strangulation of small intestines. 4 We are in favor of a congenital pathogenesis in our case as (1) the defect was located in the distal ileal mesentery near the Treves' field where the congenital defects often appear, (2) there was no past history of any trauma or surgery, and (3) the operative findings revealed no adhesions or signs of trauma/inflammation around the defect. However, hemorrhagic ascites contradict the above findings and a trivial trauma leading to a mesenteric tear that is plugged by the bowel remains a possibility.

An accurate preoperative diagnosis is often challenging because of the paucity of specific clinical signs and poor sensitivity of any radiological investigations. Blachar et al noted that findings of peripherally located small bowel lateral to the colon (a reversal from the normal pattern) and lack of omental fat between the loops and the anterior abdominal wall might be the most helpful CT signs for diagnosing transmesenteric hernias. 5 Mesenteric vessels abnormalities including displacement of the superior mesenteric artery to the right, twisting, or engorgement of the vessel might also be seen. 1 In our patient, radiological investigations failed to show evidence of internal herniation.

A lack of a hernia sac allows large length of the bowel to herniate through the mesenteric defect. This along with a small aperture of the defect predisposes transmesenteric hernias to develop volvulus and strangulation or ischemia. A prompt surgical intervention with reduction in the bowel loops, resection/anastomoses of devitalized bowel, and closure of the mesenteric defect may provide good outcomes. 6 It may be necessary to enlarge the defect for the ease of reduction in the herniated bowel, but care must be taken to avoid injury to the vessels near the edge of the defect. Closure of the mesenteric defect is must and is achieved by taking interrupted sutures with a nonabsorbable material while safeguarding the surrounding vessels.

In our case, SMV compression secondary to the internal hernia led to venous congestion in entire bowel within the SMV territory that was reversed completely on reducing the hernia. This further highlights the need of a timely surgical intervention to prevent bowel ischemia and infarction.

## Conclusion

In the absence of a past surgical or trauma history, a congenital transmesenteric hernia is an extremely rare cause of small bowel obstruction in adults. Although a preoperative diagnosis is challenging, a high index of suspicion and a prompt surgical intervention based on clinical findings are required to avert bowel ischemia and to provide a good clinical outcome. The entire mesentery needs to be evaluated and all mesenteric defects need to be sutured to prevent recurrence.
